# Thiophenone Attenuates Enteropathogenic *Escherichia coli* O103:H2 Virulence by Interfering with AI-2 Signaling

**DOI:** 10.1371/journal.pone.0157334

**Published:** 2016-06-16

**Authors:** Ingun Lund Witsø, Håkon Valen Rukke, Tore Benneche, Anne Aamdal Scheie

**Affiliations:** 1 Department of Oral Biology, Faculty of Dentistry, University of Oslo, Oslo, Norway; 2 Nordic Institute of Dental Materials (NIOM), Oslo, Norway; 3 Department of Chemistry, Faculty of Mathematics and Natural Sciences, University of Oslo, Oslo, Norway; University of Osnabrueck, GERMANY

## Abstract

Interference with bacterial quorum sensing communication provides an anti-virulence strategy to control pathogenic bacteria. Here, using the Enteropathogenic *E*. *coli* (EPEC) O103:H2, we showed for the first time that thiophenone TF101 reduced expression of *lsrB*; the gene encoding the AI-2 receptor. Combined results of transcriptional and phenotypic analyses suggested that TF101 interfere with AI-2 signalling, possibly by competing with AI-2 for binding to LsrB. This is supported by *in silico* docking prediction of thiophenone TF101 in the LsrB pocket. Transcriptional analyses furthermore showed that thiophenone TF101 interfered with expression of the virulence genes *eae* and *fimH*. In addition, TF101 reduced AI-2 induced *E*. *coli* adhesion to colorectal adenocarcinoma cells. TF101, on the other hand, did not affect epinephrine or norepinephrine enhanced *E*. *coli* adhesion. Overall, our results showed that thiophenone TF101 interfered with virulence expression in *E*. *coli* O103:H2, suggestedly by interfering with AI-2 mediated quorum sensing. We thus conclude that thiophenone TF101 might represent a promising future anti-virulence agent in the fight against pathogenic *E*. *coli*.

## Introduction

Bacteria communicate via signal molecules either produced by the bacteria themselves, by the host organism or molecules in the environment. One such process of bacterial cell-cell signaling is quorum sensing (QS), that enables bacteria to sense and respond according to cell population density, and to regulate virulence gene expression [1, 2]. Interference with QS may provide novel anti-virulence strategies to fight pathogenic bacteria [3]. The autoinducer-2 (AI-2) QS molecule is one of the most extensively studied, and AI-2 has been recognized as an intra- and inter-species communication signal. The AI-2 synthase, LuxS, is encoded by *luxS* homologues found in several different bacterial species [4–6]. The substrate of LuxS is S-Ribosylhomocysteine, which is cleaved to yield homocysteine and 4, 5-dihydroxy-2, 3- pentandione (DPD). DPD cyclizes spontaneously to form AI-2 [7].

In *Escherichia coli*, AI-2 binds the periplasmic receptor LsrB at the bacterial envelope [8, 9]. This initiates uptake of AI-2 via the AI-2 transporter formed by two transmembrane proteins, LsrC and LsrD, and an ATPase, LsrA that provides energy to the AI-2 transport. Intracellular AI-2 is phosphorylated by a kinase, LsrK. The *lsr* operon is repressed by the *lsr* repressor; LsrR. Phosphorylated AI-2 inactivates LsrR resulting in transcription of *lsr-*regulated genes and subsequent increased uptake of AI-2. Thus, AI-2 regulates its own uptake.

In *E*. *coli*, AI-2 regulates the virulence factors involved in biofilm formation and motility [10]. Adhesion is a prerequisite for bacterial colonization of both abiotic and biotic surfaces [11]. Studies have shown increasing adhesion of pathogenic *E*. *coli* to epithelial cells induced by AI-2 signaling [12, 13].

In addition to AI-2, other autoinducers such as AI-3 have been identified in *E*. *coli* [14]. AI-3 regulates gene expression through the two-component signaling system QseBC [14], with QseB being the regulator and QseC the sensor kinase [15]. Homologues of QseC are found in many important human and plant pathogens [16, 17], suggesting an important evolutionary role. Prokaryotes and eukaryotes have coexisted for millions of years, and have consequently co-evolved to sense and respond to each other`s signaling molecules [18]. *E*. *coli* responds to hormones like epinephrine and norepinephrine through QseC located in the membrane [14, 19, 20]. QseC acts as an adrenergic receptor that activates virulence genes in response to inter-kingdom cross signaling [16, 21]. Both epinephrine and norepinephrine have been shown to enhance growth and virulence in *E*. *coli* [22], and to increase motility and adhesion to HeLa cells by *E*. *coli* EHEC O157:H7 [23]. Because of the involvement of epinephrine and norepinephrine in bacterial signaling, we assessed possible interference of thiophenone with this signaling.

Several different chemical compounds have been identified as quorum sensing inhibitors (QSI) e.g. halogenated furanones [24–27]. Furanones were isolated from the red macroalgae *Delisea pulchra* and were discovered due to their capacity to inhibit bacterial growth and biofilm formation [28] by interference with AI-2 signaling [29, 30]. Sulphur analogues of furanones, thiophenones [31], have been shown to effectively inhibit biofilm formation in various bacteria, including *Staphylococcus epidermidis*, *E*. *coli* and *Vibrio harveyi* [32–35], at non-toxic concentrations [33].

We have previously shown that biofilm formation and motility in *E*. *coli* O103:H2 are reduced by both furanone F202 [35, 36], and its sulfur analogue thiophenone TF101 [35], with TF101 being the most efficacious [35]. Reduced motility by TF101 was explained by interference with the flagella synthesis, through reduced expression of flagella genes (*flhD*) [35], genes that are regulated by AI-2 [15].

This study aimed to elucidate the mechanisms of action of the quorum sensing inhibitor TF101 in *E*. *coli* O103:H2. The hypothesis was that TF101 interferes with virulence factors such as adhesion and biofilm formation regulated by AI-2, epinephrine or norepinephrine.

## Materials and Methods

### Thiophenone

**Fig 1:**
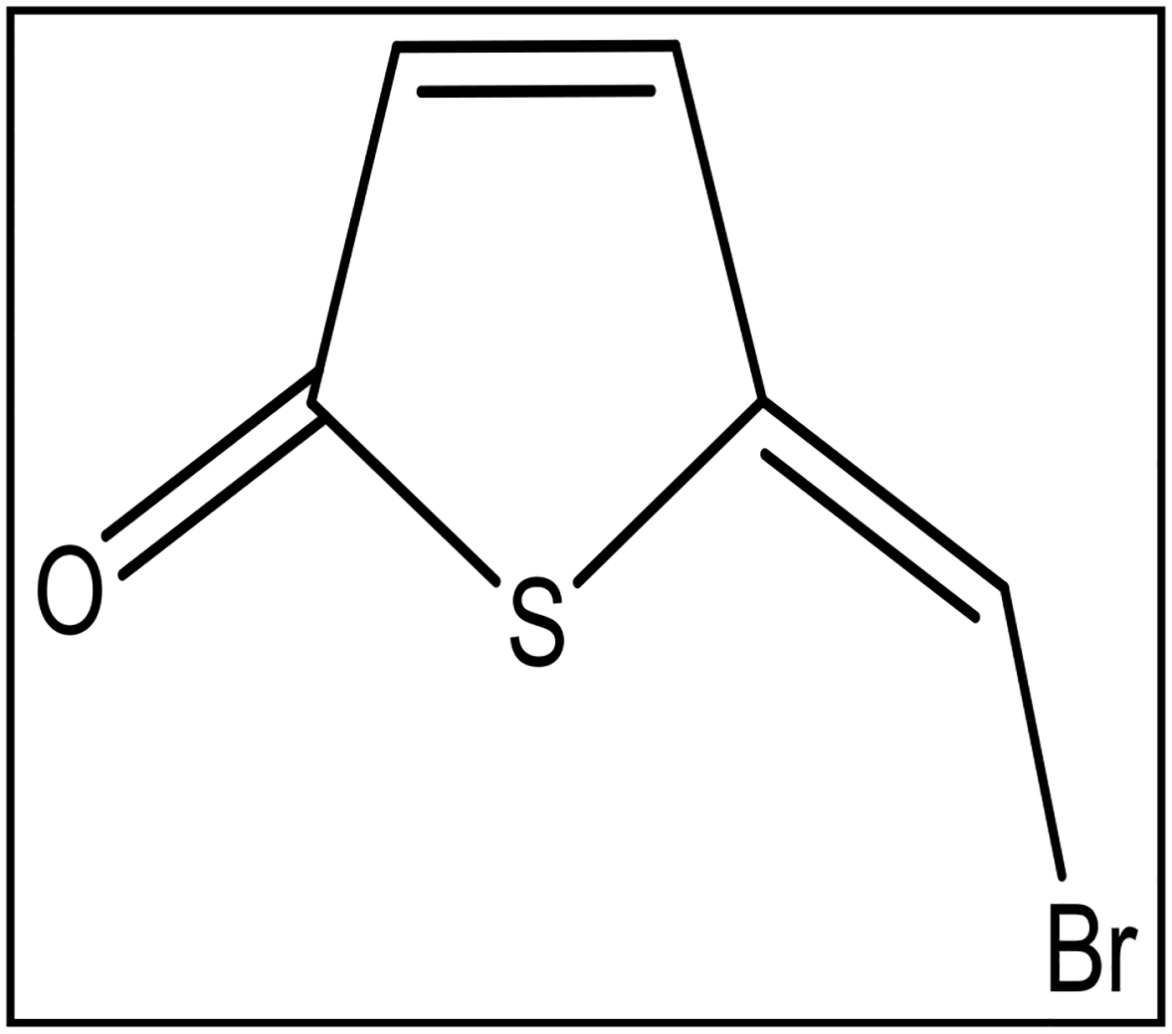
The chemical structure of (Z)-5-(bromomethylene)-thiophene-2(5H)-one (TF101).

Thiophenone TF101, (*Z*)-5-(bromomethylene) thiophen-2 (5*H*)-one (Fig 1), was synthesized as reported previously [31]. Thiophenone was dissolved in 70% ethanol at 50 mM and stored at −20°C.

### AI-2

Synthetic DPD ((S)-4, 5-dihydroxy-2, 3-pentanedione, OMM Scientific Inc., TX, USA) was the source of AI-2 used.

### Bacterial strains and culture media

Atypical enteropathogenic (aEPEC) *Escherichia coli* O103:H2 strain 2006-22-1153, isolated from sheep was used as a model organism in this study (Table 1). The strain was verified and characterized at the National Reference Laboratory at the Norwegian Veterinary Institute. *E*. *coli* ABU83972 (OR: K5:H^−^, *lsr*^−^), originally isolated from a young Swedish girl [37], was included in the biofilm assay. The strains were stored at -80°C in LB broth (Difco) supplemented with 15% glycerol, and recovered on LB agar plates (bacto-trypton 10 g/L, yeast extract 5 g/L, agar 15 g/L) at 37°C overnight. The bacterial cultures were transferred into LB broth and incubated with shaking at 37°C for 5 h to obtain working cultures. For biofilm experiments, *E*. *coli* O103:H2 was grown in LB without NaCl (bacto-trypton 10 g/L, yeast extract 5 g/L) [38], hereafter called LB^b^, whereas *E*. *coli* ABU83972 was grown in LB broth (Difco). The differences in the growth media used in the biofilm experiments are due to differences in the preferred growth conditions in the two strains.

**Table 1:** Bacterial strains used in this study.

*E*. *coli* strain	Characteristics	Phenotype	Reference
2006-22-1153 (1153)	Enteropathogenic *E*. *coli* O103:H2	*Stx1*^*-*^, *stx2*^*-*^, *eae*^*+*^	[52]
83972	ABU isolate (OR:K5:H^−^)	*lsr* ^*-*^	[37]

### Sample preparation, RNA isolation and qPCR

Overnight culture of *E*. *coli* O103:H2 was diluted in LB broth to OD_600_ = 0.01, and incubated with shaking at 37°C. When the culture reached OD_600_ = 0.5, 10 μM TF101, 10 μM AI-2, or 10 μM TF101 and 10 μM AI-2 in combination, was added. Bacteria in plain LB were included as control. The cultures were allowed to grow, and pellets were collected every hour by centrifugation (2000g x 4°C x 5min) and stored at– 80°C. Total RNA was isolated from harvested *E*. *coli* using the High Pure RNA isolation kit (Roche Applied Science, Mannheim, Germany) according to the manufacturer`s protocol. In addition to the DNase treatment included in the RNA isolation protocol, an additional DNase treatment was performed using Turbo DNase (Thermo Fisher Scientific Inc.). cDNA was synthetized using MMLV Reverse transcriptase 1^st^-strand cDNA Synthesis Kit (Epicenter Biotechnologies) according to the manufacturer`s protocol. The primer pairs used are listed in Table 2. Real time reactions were performed using the Thermo Scientific Maxima SYBR Green/ROX qPCR Master Mix (ThermoScentific), and real-time amplification was carried out using the Stratagene Mx3005 P Multiplex Quantitative PCR systems (Stratagene, La Jolla, CA). The gradient thermocycling program was set for 40 cycles at 95°C for 15 s, 59°C for 30 s, and 72°C for 30 s, with an initial cycle at 95°C for 10 min. The data were collected and analyzed by normalization against the housekeeping gene *rpoA* using the MxPro software.

**Table 2:** Primers used in this study.

ILW009_fimH	Forward	5`-atattgctgagtccacccgc-3`
ILW009_fimH	Reverse	5`-ttgcgtccaagtaccaccag-3`
ILW004_rpoA	Forward	5`-caaccattctggctgaacaa-3`
ILW004_rpoA	Reverse	5`-gcggacagtcaattccagat-3
ILW001_lsrB	Forward	5`-cggagtgccgctcttactac-3`
ILW001_lsrB	Reverse	5`-gtaacggtggggcttgagta-3`
ILW010_eae	Forward	5`-actgtggctcgatttgctga-3`
ILW010_eae	Reverse	5`-ctccgattcctctggtgacg-3`

### Protein ligand interaction *in silico*

The protein ligand binding of AI-2 and TF101 in LsrB (LsrB from Salmonella typhimurium; PDB; http://www.rcsb.org/pdb/) [39] was predicted by using PyRx virtual screening tool (ver 0.9.2), followed by a visualization of the interaction using PyMol (ver 4.0).

### Adherence to epithelial cells

The colorectal adenocarcinoma cell line Caco-2 was used as epithelial cells. The cells were grown in RPMI- 1640 (Sigma-Aldrich) with 2 mM L-glutamine (Sigma-Aldrich), 10% FBS and 1% Antibiotic Antimitotic Solution (Sigma-Aldrich), in 5% CO_2_ at 37°C. Following trypsination, the cells were washed once in complete RPMI- 1640, and 1 mL seeded at a concentration of 4 x 10^5^ cells per mL in 24 well plates (Nunc, Thermo Fisher Scientific) and grown to confluence.

*E*.*coli* O103:H2 incubated with aeration overnight in LB^b^ medium was washed in PBS before centrifugation (5000g x 4°C x 5 min). The bacteria were re-suspended in RPMI-1640 without antibiotics and added to confluent Caco-2 cells in 24- well plates, to a multiplicity of infection (MOI) of 40:1. TF101 was added (10 μM final concentration) to assess the effect of TF101 on adhesion. Epinephrine (50 μM final concentration, Sigma-Aldrich, USA), norepinephrine (50 μM final concentration, Sigma-Aldrich, USA), or AI-2 (10 μM final concentration) was added in triplicate wells to assess their effect on adhesion. The plates were incubated for 4 h, 37°C. The cells were then washed twice with PBS to remove non-adherent *E*. *coli* and the Caco-2 cells were lysed with 0.1% Triton-X100. The lysates were diluted and plated on LB agar plates for CFU counts. Cells infected with bacteria without chemicals were included as negative control. To confirm that TF101 had no effect on receptors on the Caco-2 cells, the cells were exposed to TF101 prior to the adhesion assay in separate experiments. Fresh RPMI medium with TF101 (10 μM final concentration) was added to the cells. The cells were incubated at 37°C (5% CO_2_) for 60 minutes. The medium with TF101 was removed; the cells washed twice with PBS to remove any remaining TF101, and fresh RPMI was added to each well. The bacteria were added to the cells and adhesion was quantified as described above.

Potential bacterial invasion was assessed using gentamicin protection assay as previously described [40], with some modifications. After the infection period described above, the Caco-2 cells with adherent *E*. *coli* were washed twice with PBS and fresh RPMI with gentamycin (50 mg/ L) was added to the wells to kill extracellular bacteria. The MIC of gentamycin was determined as 6 mg/L prior to the invasion experiment. RPMI without antibiotics was added to control wells. After 1 h incubation, the wells were washed four times with PBS to remove antibiotic residues. The cells were lysed by 0.1% Triton-X100. The lysates were serially diluted and plated on LB plates as above for CFU count.

Scanning electron microscopy (SEM) was used to visualize adherent *E*. *coli* on Caco-2 cells. Caco-2 cells were grown to confluence on polystyrene coverslips (Nunc Thermanox Coverslips, Thermo Scientific, Rochester, NY) with adherent *E*. *coli* as described above. The samples were fixed with 2.5% glutaraldehyde in 0.1 M Sørensen phosphate buffer and stored at 4°C until processed and examined by SEM (model XL 30 ESEM, Philips, Eindhoven) as described previously [30].

### Cytotoxic effect of thiophenone

A possible cytotoxic effect of TF101 on Caco-2 cells was assessed by using the lactate dehydrogenase (LDH) release assay (CytoTox 96 Non-Radioactive Cytotoxicity Assay kit; Promega, Madison, WI). Caco-2 cells were cultured as described previously. A total of 50 000 cells/ mL were seeded in flat-bottom 24-well polystyrene microtiter plates (Nunc) and grown to confluence. The growth media were discarded and the Caco-2 cells were exposed to different concentrations of thiophenone TF101 (0 μM, 2.5 μM, 5 μM, 10 μM or 50 μM final concentration) dissolved in fresh RPMI. The cells were further incubated for 4 h, before the absorbance of the supernatant was measured (490 nm) according to the manufacturer`s protocol using the Synergy HT Multi-Detection Microplate Reader (Biotek).

### Biofilm formation

The effect of TF101 on biofilm formation by *lsrB* proficient (*E*. *coli* O103:H2, *lsr*^+^) and non-proficient (*E*. *coli* ABU83972, *lsr*−) strains was assessed and compared. Overnight cultures in LB medium were diluted 1:1000 in fresh LB^b^ for *E*. *coli* O103:H2, and LB for *E*. *coli* ABU83972, incubated with aeration at 37°C for 5 h and diluted 1:200 in the respective fresh media (OD_600_ = 0.02). To assess the effect on biofilm formation, TF101, AI-2, epinephrine or norepinephrine was added to the bacterial suspensions of *E*. *coli* O103:H2 at final concentrations of 10 μM, 10 μM, 50 μM or 50 μM, respectively. In order to investigate possible interference of TF101 with the different signaling systems, 10 μM TF101 was added simultaneously with 10 μM AI-2, and 50 μM of epinephrine or norepinephrine, in the biofilm assay.

The effect of TF101 and AI-2 on the ABU83972 (*lsr*^−^strain) was tested by adding TF101 to the cultures at final concentrations of 5 μM, 10 μM or 50 μM, and AI-2 at a final concentration of 10 μM. Samples of 200 μL were added to flat-bottom, 96-well polystyrene microtiter plates (Nunc, Thermo Fisher Scientific). The plates were incubated statically for 48 h at 20°C for *E*. *coli* O103:H2 and overnight at 37°C for ABU83972, according to growth conditions required by the respective strains. Biofilm quantity was assessed after removing the planktonic cells by inverting the plates and washing the wells twice with 0.9% NaCl. Adherent cells were stained with 0.1% safranin solution for 30 min, followed by washing at least three times with 0.9% NaCl. The safranin stain was released with 30% acetic acid, and OD_530_ nm was measured (Synergy HT Multi-Detection Microtiterplate Reader, Biotek, VT). The assay was performed in six parallels, and the experiment was repeated twice. The biofilm mass was calculated as % of control.

### Statistical analysis

All experiments were performed as minimum two independent experiments with at least three parallels of each sample, using freshly prepared reagents. One-way ANOVA followed by Student- Newman- Keuls method was used for the comparisons in the adhesion test and the biofilm analysis involving the effect of TF101 and epinephrine/ nor-epinephrine, and the differences in gene expression. The effect of AI-2 on biofilm formation was determined using t-test. For all statistical analyses, the level of statistical significance was set at *P* < 0.05.

## Results

### Thiophenone interferes with AI-2 signaling

**Fig 2:**
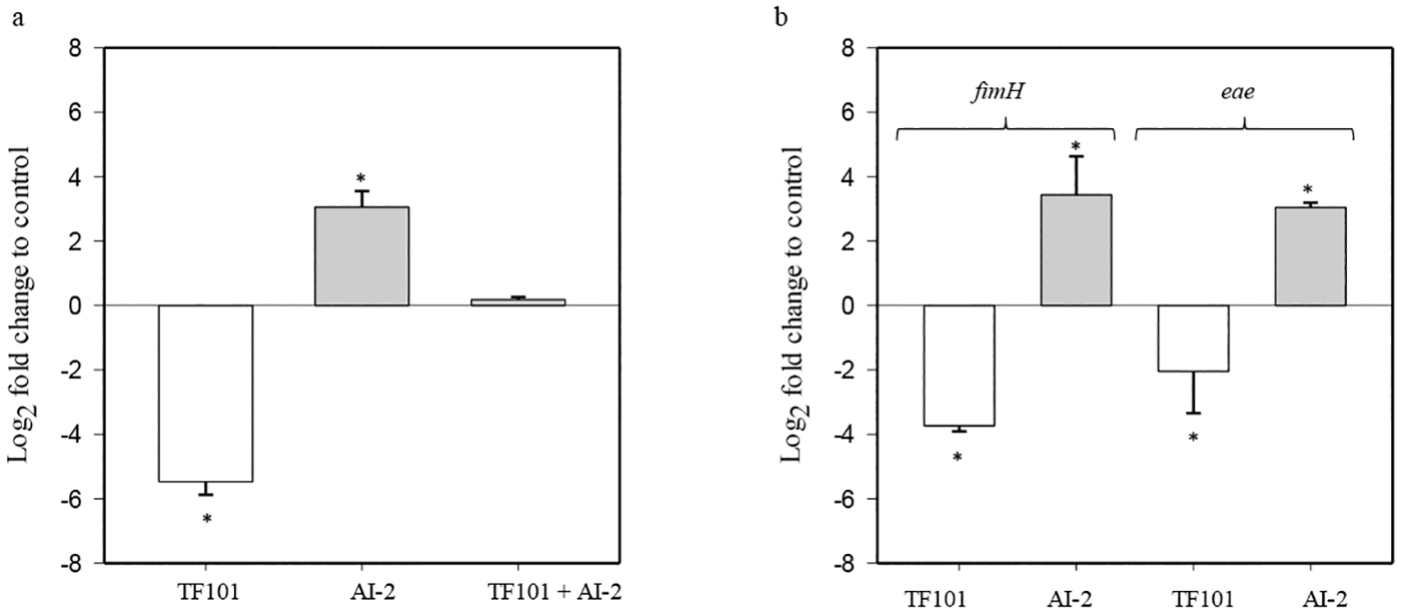
The effect of TF101 or AI-2 on expression of *lsrB*, *fimH* and *eae* in *E*. *coli* O103:H2. (a) TF101 significantly reduced expression of *lsrB*, while AI-2 gave a significant increase in expression of *lsrB*. TF101 and AI-2 added simultaneously attenuated the increase in *lsrB* expression following AI-2 stimulation (b) Expression of *fimH* and *eae* was significantly decreased following exposure to TF101, while AI-2 gave a significant increase in the expression of the same genes. (0 = control without exposure to TF101, AI-2 or both, respectively). The data are presented as mean values ± SD (n = 6). *Significantly different from control (*P* < 0.05).

The possible interference of thiophenone with the AI-2 signaling system was investigated by measuring expression of the *lsrB* gene, encoding the AI-2 binding receptor, using quantitative real-time PCR (qPCR) with samples collected after 2 hours of exposure to AI-2, TF101 or AI-2 and TF101 in combination. Exposure of *E*. *coli* to 10 μM TF101, reduced expression of *lsrB*, while addition of AI-2 gave a significantly increased expression of *lsrB* (*P* < 0.01). The increase in gene expression in response to AI-2, was attenuated by TF101 (Fig 2a).

**Fig 3:**
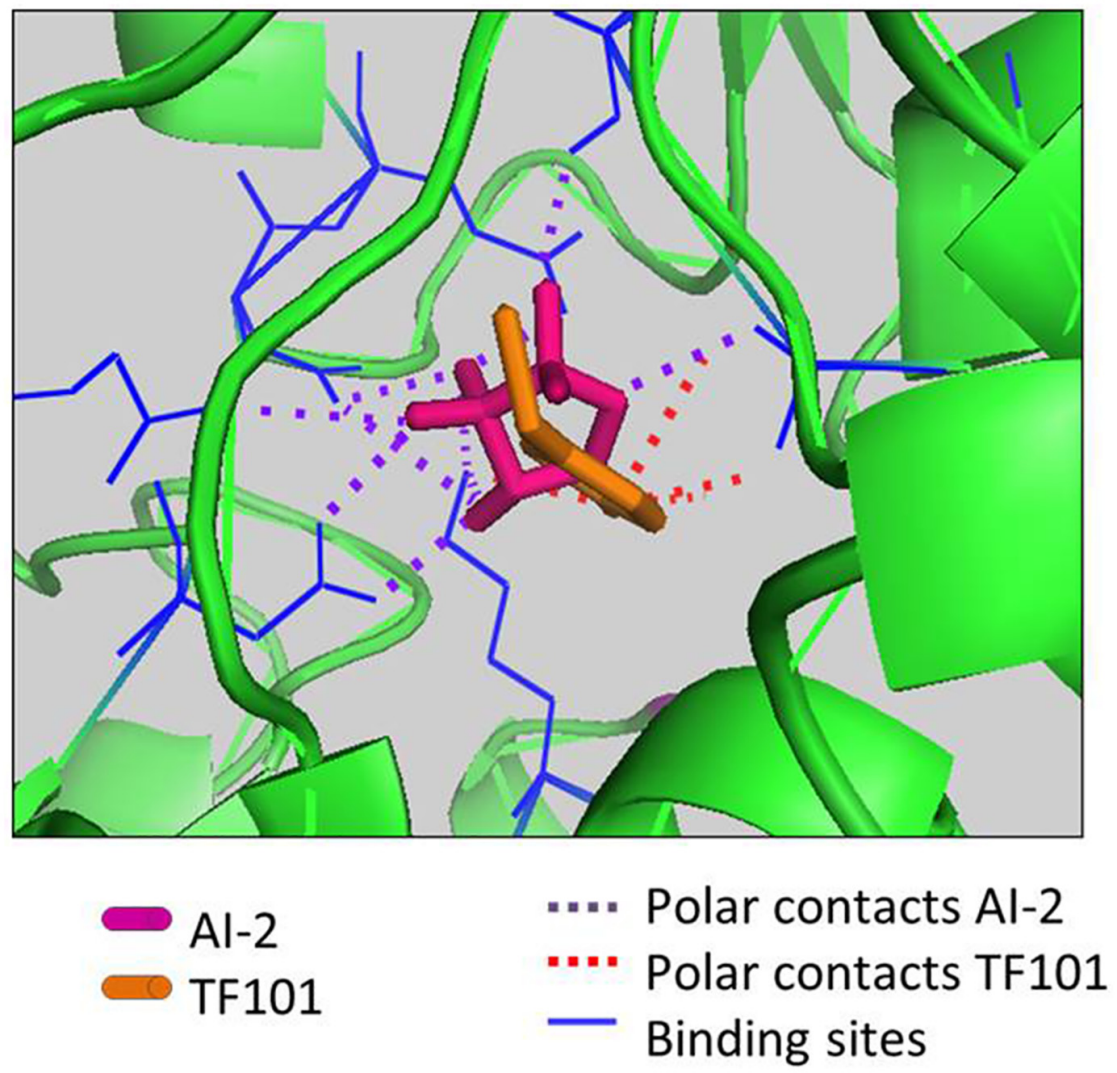
*In silico* interaction of TF101 and AI-2 with the LsrB receptor. *In silico* analyses predicted possible protein ligand interaction of TF101 and the LsrB receptor, indicating that TF101 might act as a competitive antagonist for the AI-2 receptor.

The binding affinity of TF101 to LsrB receptor was predicted using PyRx/Autodock Vina software. The program predicted binding of TF101 binding sites in the AI-2 binding pocket of the LsrB receptor from Salmonella (Fig 3). Pairwise amino acid alignment of *lsrB* from *E*. *coli* and Salmonella are found in the supplementary material (S1 Fig). The predicted binding of AI-2 was estimated to be -7.1 ^kcal^/_mol_, while the binding affinity of TF101 was -4.2 ^kcal^/_mol_.

### Thiophenone TF101, AI-2, epinephrine and norepinephrine affect adherence to epithelial cells

**Fig 4:**
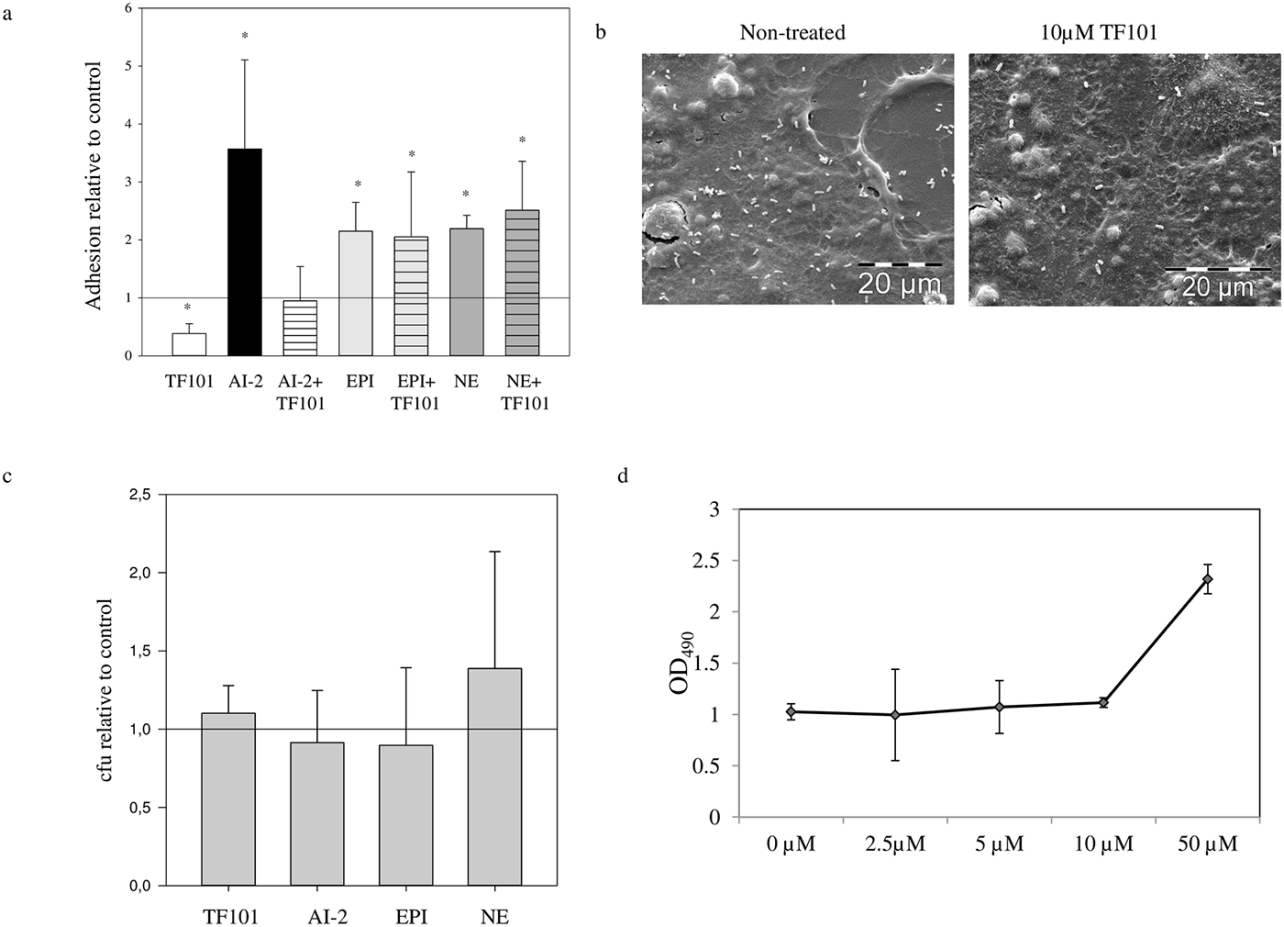
Adhesion of *E*. *coli* O103:H2 to Caco-2 cells. (a) Mean ± SD number of bacteria (n = 12) attached to Caco-2 cells, relative to control (= 1 reference line). 10 μM TF101, 10 μM AI-2, 10 μM AI-2 + 10 μM TF101, 50 μM epinephrine (EPI), 50 μM epinephrine + 10 μM TF101, 50 μM norepinephrine (NE), or 50 μM norepinephrine + 10 μM TF101 was added during the adhesion assay. *Significantly different from control, *P* < 0.05. (b) SEM images of adherent *E*. *coli* O103:H2 on Caco-2 cells with or without addition of 10 μM TF101. Scalebar, 20 μm. (c) Adhesion of *E*. *coli* O103:H2 to Caco-2 cells pre-exposed and non-pre-exposed to TF101. The cells were exposed to 10 μM TF101 before the bacteria were added in order to test any possible effect of TF101 on the cells. The effect of pre-exposure of TF101 is presented relative to non-pre-exposed cells (indicated by the reference line = 1). The data are presented as mean values ± SD (n = 12). (d) Cytotoxic effect of TF101 on Caco-2 cells. Release of lactate from Caco-2 cells exposed to 0 μM, 2.5 μM, 5 μM, 10 μM and 50 μM T101. The data are presented as mean values ± SD (n = 6).

Adhesion of *E*. *coli* to epithelial surfaces is the first step in colonization; we therefore investigated how thiophenone may interfere with AI-2 and inter-kingdom signaling molecules involved in *E*. *coli* adhesion. *E*. *coli* exposed to 10 μM TF101 showed a 2.6 fold reduction in adhesion, assessed by CFU compared to samples without TF101, while exposure to AI-2 increased adhesion 3.5 fold. Interestingly, TF101 attenuated the adhesion-enhancing effect of AI-2, suggesting that TF101 interacted with AI-2 signaling (Fig 4a). Scanning electron microscopy images confirmed the reduced adhesion of *E*. *coli* O103:H2 to Caco-2 cells, when exposed to TF101 compared to control (Fig 4b). The concentration of TF101 used did not affect planktonic growth in RPMI medium (data not shown). In addition, there was no difference in bacterial adhesion to Caco-2 cells when the cells were exposed to TF101 prior to the adhesion assay (Fig 4c). To investigate whether difference in adhesion could be attributed to a cytotoxic effect of TF101, cytotoxicity against Caco-2 cells was assessed by the LDH release assay. There was no difference in LDH release from Caco-2 cells exposed to TF101 at concentrations from 10 μM and lower, indicating no cytotoxic effect against Caco-2 cells at these concentrations (Fig 4d).

AI-2 has been shown to alter expression of genes encoding fimbria and flagella. We therefore investigated whether TF101 affected expression of the *fimH* and *eae* genes, encoding type 1 fimbria and intimin respectively. The *fimH* and *eae* genes showed significantly (*P* < 0.05) reduced expression upon exposure to 10 μM TF101, while exposing *E*. *coli* to AI-2 led to increased expression (*P* < 0.05) (Fig 2b).

To investigate whether TF101 also attenuated adhesion by other signaling molecules mediating adhesion, exposure to the inter-kingdom signaling molecules epinephrine and norepinephrine was performed. Epinephrine (50 μM), and norepinephrine (50 μM) increased adhesion to Caco-2 cells by 2.1 and 2.2 fold respectively, however TF101 did not attenuate the stimulatory effect on adhesion (Fig 4a).

The mean level of bacterial invasion in infected cells was close to nil (data not shown). The effect of TF101, AI-2, epinephrine or norepinephrine on invasion was therefore not investigated.

### Effect of AI-2, TF101, epinephrine and norepinephrine on biofilm formation

**Fig 5:**
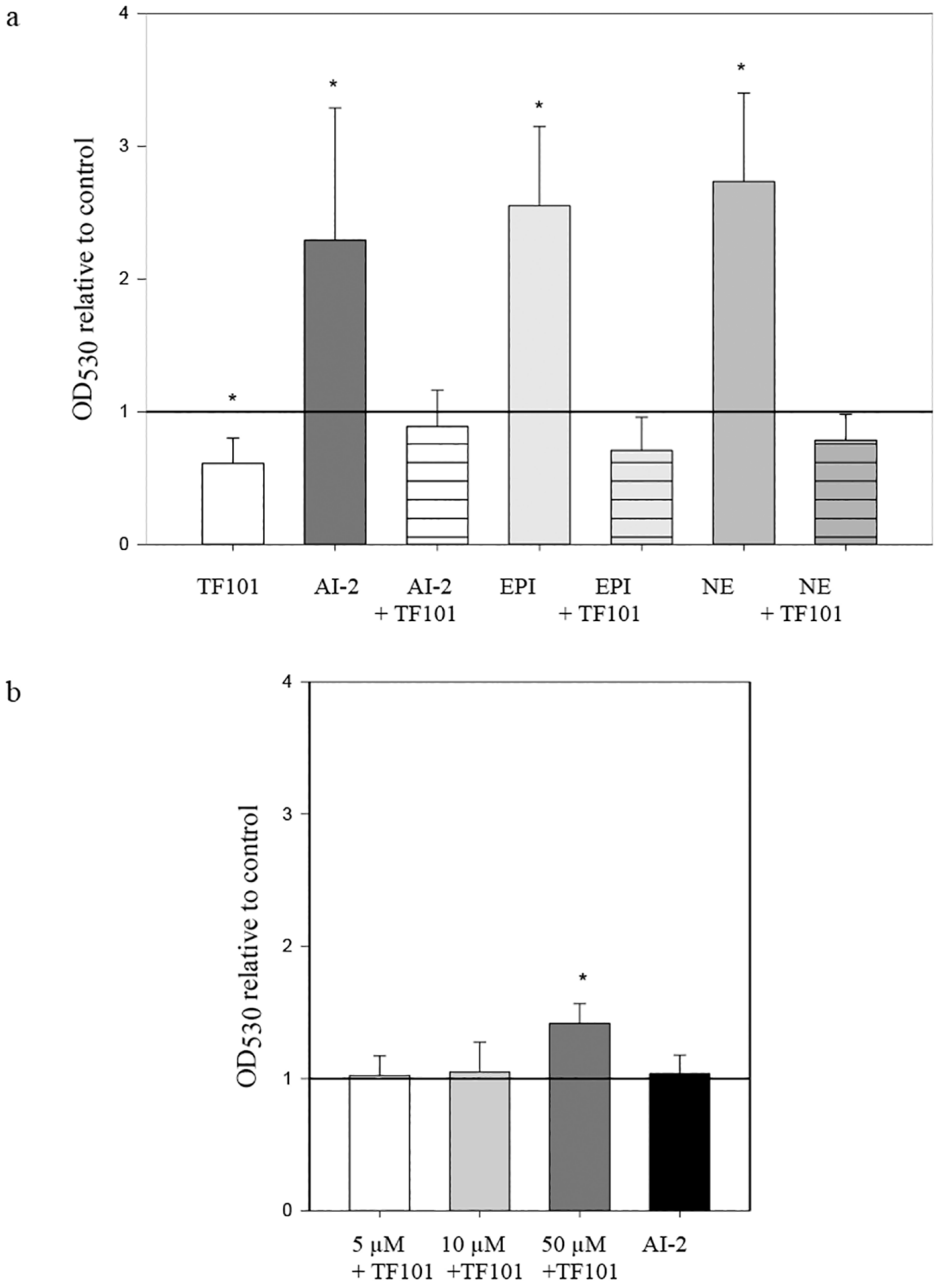
Biofilm formation. (a) Relative effect of TF101, AI-2, epinephrine (EPI) or norepinephrine (NE) on biofilm formation by *E*. *coli* O103:H2. 10 μM TF101, 10 μM AI-2, 10 μM AI-2 + 10 μM TF101, 50 μM EPI, 50 μM EPI + 10 μM TF101, 50 μM NE, or 50 μM NE + 10 μM TF101 was added during the biofilm assay. The effect of the different chemicals is presented as relative to control value (reference line (= 1)). The data are presented as mean values ± SD (n = 10). *Significantly different from control (*P* < 0.05). (b) Relative effect of TF101 or AI-2 on biofilm formation by E. *coli* ABU 83972 (*lsr*^-^). In *E*. *coli* ABU83972, neither TF101 nor AI-2 affected biofilm formation, except at 50 μM TF101 which gave a significant increase. The effect of TF101 or AI-2 on biofilm formation is presented as relative to control (= 1 reference line). The data are presented as mean values ± SD (n = 10). *Significantly different from control (*P* < 0.05).

TF101 reduced biofilm formation by *E*. *coli* O103:H2 significantly (*P* < 0.05) at 10 μM, while AI-2 increased biofilm formations by 2.3 fold. By adding TF101 and AI-2 simultaneously, TF101 attenuated the enhancing effect of AI-2 on biofilm formation (Fig 5a). Epinephrine and norepinephrine increased biofilm formation by 2.5 and 2.7 fold, respectively, while TF101 diminished the stimulatory effect on biofilm formation (Fig 5a). Epinephrine and norepinephrine did not affect planktonic bacterial growth (S2 Fig). To further investigate the specificity of TF101 interference with AI-2 signaling, the *lsr* negative strain *E*. *coli* ABU83972 was used. Biofilm formation by this strain was unaffected by TF101 at 5 μm and 10 μm. However, 50 μm TF101 significantly enhanced biofilm formation in this *lsr* negative *E*. *coli* strain (*P* < 0.05) (Fig 5b), while addition of AI-2 did not stimulate biofilm formation.

## Discussion

In this study, we investigated how thiophenone TF101 interfered with AI-2 quorum sensing mediated regulation of virulence in *E*. *coli* O103:H2 (EPEC). We furthermore explored how the host-derived hormones, epinephrine and norepinephrine, affected adhesion and biofilm formation alone and in combination with thiophenone.

The exact mechanism of action of TF101 in *E*. *coli* is still not fully elucidated, but the present results give support to our hypothesis that TF101 interacts with the AI-2 controlled *lsr* operon and possibly competes for the LsrB receptor. The effect of TF101 on *lsrB* expression was studied to determine whether TF101 interferes with AI-2 signaling. The expression of *lsrB* was significantly reduced in response to TF101. The importance of AI-2 in the activation of *lsr* genes was confirmed by the upregulation of *lsrB* in presence of AI-2. Reduced expression of *lsrB* could consequently lead to a disruption in AI-2 internalization, resulting in down-regulation of AI-2 regulated genes involved in virulence. By exposing the bacteria to TF101 and AI-2 simultaneously, we showed that the upregulation of *lsrB* in response to AI-2 was diminished.

The *in silico* AI-2 and TF101 docking results predicted binding of TF101 in the AI-2 binding pocket of the LsrB receptor, suggesting that TF101 may act as a competitive antagonist for the AI-2 receptor in *E*. *coli*. AI-2 activates transcription of the *lsr* operon after phosphorylated AI-2 interacts with LsrR, which then relieves its repression of the *lsr* operon. The present results suggest that TF101 could act by distrupting AI-2 activity, and consequently inactivate the *lsr* operon and reduce AI-2 internalisation.

TF101 has previously been shown to reduce expression of *flhD* [35], the master regulator of flagella synthesis, which is regulated by AI-2 [29, 41]. TF101 has also been shown to decrease the DNA- binding activity of the master regulator LuxR in *V*. *harveyi* [34]. To our knowledge, our study is the first to show that TF101 interfered with expression of a gene that is directly regulated by AI-2 signalling. This further supports the hypothesis that TF101 may function through interference with AI-2 mediated gene regulation. Consistent with this are the previous reports showing that TF101 interfered with AI-2 induced bioluminescence in the marine pathogen *Vibrio harveyi*, and that TF101 does not interfere with AI-2 synthesis [34, 35]. Further to test whether TF101 interferes with AI-2 signaling, we exposed *E*. *coli* simultaneously to TF101 and AI-2 and assessed subsequent adhesion to Caco-2 cells and biofilm formation. We found that TF101 attenuated the enhancing effect of AI-2 on adhesion and biofilm formation, thus supporting the assumption that TF101 interfered with AI-2 signaling and its ability to activate genes involved in adhesion and biofilm formation. The *in silico* analysis predicted that TF101 migth bind to the LsrB receptor, and thus inhibit binding of AI-2. We thus propose that TF101 could function as a competitive antagonist, preventing AI-2 internalization and *lsr* operon activation.

Thiophenone TF101 reduces biofilm formation and motility in *E*. *coli* O103:H2 [35], possibly by interacting with AI-2 and *lsr* activity. This hypothesis was further explored by assessing the effect of TF101 on biofilm formation in *E*. *coli* ABU83972, a recognized good biofilm former [42] lacking the *lsr* operon [43]. While *E*. *coli* ABU83972 did not respond to TF101 at concentration expected to reduce biofilm formation, our results also showed that AI-2 did not stimulate biofilm formation in *E*. *coli* ABU 83972. Interestingly, 50 μM, a concentration that normally is toxic to bacteria, resulted in increased biofilm. With the lack of *lsr* genes, *E*. *coli* ABU 83972 appears to regulate biofilm formation in a non AI-2 dependent manner. Notably, *E*. *coli* O103:H2 and ABU83972 required different experimental conditions to form biofilm. Nevertheless, using two *E*. *coli* strains with different *lsr* status to study the effect of TF101, could give some clues of the mechanism of action of TF101 and its possible interference with AI-2 signaling in *E*. *coli*. As the results suggest that the *lsr* genes could be involved in the mechanism of action of TF101 in *E*. *coli* O103:H2, LsrB could be an attractive drug target.

Several studies have characterized different adhesion patterns of EPEC to epithelial cells [44, 45]. According to the criteria stated in these reports we identify the adherence patterns of *E*. *coli* O103:H2 in this study, as IS (isolated bacteria) pattern; few isolated individual bacteria over the cells. (Fig 4b) [44]. The role of AI-2 mediated signaling in adhesion of *E*. *coli* to epithelial cells, has previously been explored only briefly. Bansal *et al* showed that AI-2 increased adhesion of *E*. *coli* O103:H2 EPEC strain to HeLa cells at concentrations of 100 and 500 μM [46]. However, in our study we show that an AI-2 concentration of 10 μM significantly increased adhesion to Caco-2 cells. Even though the cell line and media used in the two studies were different, they both indicate that AI-2 may play an important role in regulating adhesion to eukaryotic cells. We furthermore showed that the quorum sensing inhibitor (QSI) thiophenone TF101 significantly reduced the adhesion. To our knowledge, this is the first study to show the effect of a QSI on *E*. *coli* adhesion to epithelial cells. The transcriptional analysis showed decreased expression of *fimH* and *eae*, encoding the adhesion factors Type 1 fimbria and intimin respectively, in response to TF101, and increased expression in response to AI-2. Type 1 fimbria is a common adhesion factor found in both commensal- and pathogenic *E*. *coli*. The most important adhesion factor in EPEC is intimin, an outer membrane protein, encoded by the *eae* gene. *Eae* is found within the pathogenicity island LEE (locus of enterocyte effacement). Intimin is responsible for early bacterial adhesion to eukaryotic cells. Tir (translocated intimin receptor) acts as the receptor for intimin, and is translocated into the eukaryotic plasma membrane via Type III secretion system (T3SS). The LEE- encoded Type III secretion systems are key virulence factors of Gram negative enteric pathogens, and serve to inject bacterial proteins directly into host cells. Altogether, these LEE-encoded factors contribute to the characteristic attaching/effacing lesions (A/E lesions) in EPEC [47].

Our results indicate that decreased adhesion to Caco-2 cells following treatment with TF101 could be explained by a reduction in the production of adhesion factors such as intimin and Type 1 fimbriae. The increase in gene expression of the adhesion factors after stimulation with AI-2 also indicates that AI-2 is involved in the regulation of these virulence factors. Our results are thus consistent with other studies reporting that several genes involved in flagellar and fimbria biosynthesis are upregulated in response to AI-2 [46, 48].

The cytotoxicity assay showed that TF101 at concentrations used in this study did not exert cytotoxic effects on Caco-2 cells. Our findings are in line with results from a previous study, showing that thiophenone TF101 at these concentrations did not affect human fibroblasts [33]. We furthermore tested whether the effect of TF101 on adhesion of *E*. *coli* to Caco-2 cells could be explained by altered surface properties of the Caco-2 cells. We exposed the cells to TF101 prior to the adhesion assay. There was no difference in adhesive capacity of *E*. *coli* O103:H2 EPEC between samples with pre-treated- versus non-pre-treated Caco-2 cells. Hence, the reduced adhesion related to TF101 could not be explained neither by cytotoxic effects of TF101 nor by alterations of the surface of the Caco-2 cells.

The mechanism of action of TF101 is still not completely revealed. In order to study whether TF101 interfered with other signaling pathways involved in regulation of virulence, we chose epinephrine and norepinephrine due to their association with enhanced bacterial growth, biofilm formation and adhesion [20, 23]. This study confirmed that *E*. *coli* O103:H2 is able to sense epinephrine and norepinephrine, which could be the first step in a sequence of events leading to infection. Several studies have suggested that epinephrine and norepinephrine act as signaling molecules between the host and the bacteria [14, 49]. Our results showed that epinephrine and norepinephrine increased adhesion to epithelial cells, and increased biofilm formation by *E*. *coli* O103:H2. This is in agreement with several other studies on the regulation of eukaryotic stress hormones in *E*. *coli* virulence and infection [16, 23, 50]. We added TF101 simultaneously with epinephrine or norepinephrine in order to test whether TF101 interfered with their role as signal molecules. From our adhesion assay we showed that TF101 did not interfere with host-bacteria interaction by interfering with epinephrine/norepinephrine. On the other hand, in the biofilm assay we observed that TF101 attenuated the biofilm-enhancing effect of epinephrine and norepinephrine. Biofilm formation and adhesion are two virulence factors regulated by different mechanisms in *E*. *coli*. It is still unclear how epinephrine and norepinephrine stimulate adhesion and biofilm formation. We could argue that the mechanisms regulating adhesion and biofilm are different, and TF101 might interfere with these mechanisms differently. None of these results give a strong indication that TF101 interfere with epinephrine/norepinephrine signaling, however it does suggest TF101 as an effective biofilm inhibitor able to attenuate the stimulating effect of epinephrine/norepinephrine and AI-2 signaling. Conversely, from our results, we cannot exclude the possibility that the effect of TF101 may not be specific to AI-2 signaling, and that other unknown mechanisms can be involved. This might be true especially for biofilm formation, but also for adhesion to epithelial cells.

Epinephrine and norepinephrine did not increase the growth rate of *E*. *coli*, a result that is in contrast to prior reports [22, 51]. However, similar growth rates irrespective of epinephrine or norepinephrine addition emphasize that the increase in adhesion and biofilm formation cannot be explained by increased cell density due to increased bacterial growth. These results may be important in order to reveal the regulation of adhesion and colonization of *E*. *coli* O103:H2.

One of the challenges with using some bactericidal compounds for treating bacterial infections is lysis of the bacteria, and the concomitant release of toxins and pro-inflammatory mediators, which may lead to tissue destruction and treatment failure. This highlights the need for drugs that are effective without lysis of the bacteria. Thiophenone might be one such drug representing a non-bactericidal anti-virulence agent, hence; endotoxins and other products will not be released. Another important benefit of using non-bactericidal anti-virulence compounds like TF101 is that it does not exert a strong selective pressure for the development of resistance.

The pathogenicity of *E*. *coli* is a complex series of events including both bacterial quorum sensing molecules and a cross talk communication with the host. The present study show that TF101 interferes with *E*. *coli* O103:H2 virulence possibly by interfering with quorum sensing, however, future studies with additional pathotypes and other bacterial species are warranted. We propose that thiophenones represent promising anti-virulence agents in the fight against pathogenic bacteria.

## Supporting Information

S1 Fig Pairwise alignment of the amino acid sequence of *lsrB* from Salmonella enterica subsp.serovar enterica typhimurium and *E*. *coli* O103:H2 str.12009. The yellow marks represent the amino acids in which AI-2 bind to in the binding pocket of the LsrB receptor.
(TIF)

S2 Fig The effect of epinephrine and norepinephrine on planktonic growth. No significant effect on planktonic growth was observed in response to epinephrine or norepinephrine.
(TIF)
