# Supplementary figures and images for: Thiophenone Attenuates Enteropathogenic Escherichia coli O103:H2 Virulence by Interfering with AI-2 Signaling
Source: PLoS One. 2016 Jun 16;11(6):e0157334. doi: 10.1371/journal.pone.0157334 (PMC4911109; doi:10.1371/journal.pone.0157334)

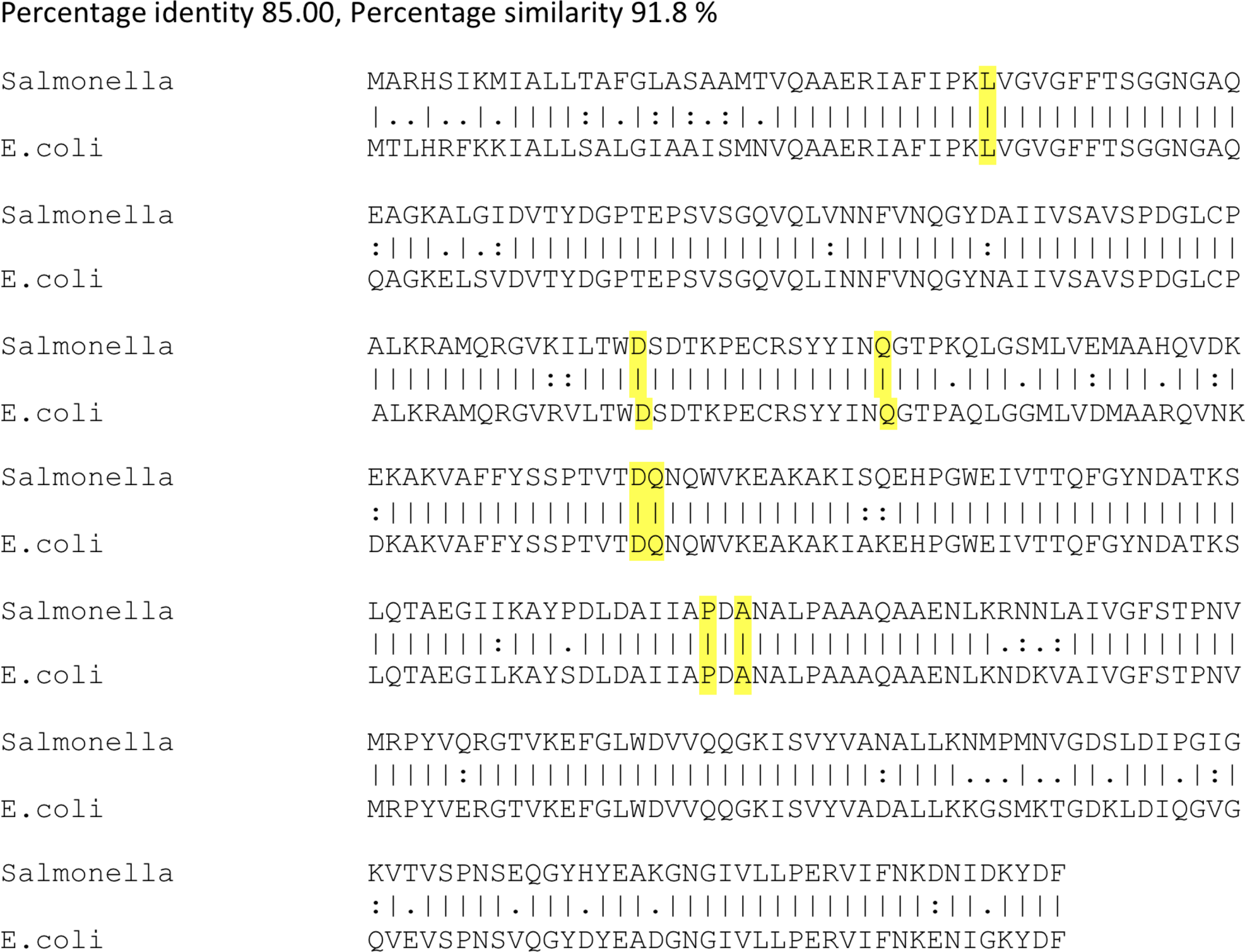

Supplement: S1 Fig — The yellow marks represent the amino acids in which AI-2 bind to in the binding pocket of the LsrB receptor. (TIF) [file pone.0157334.s001.tif]

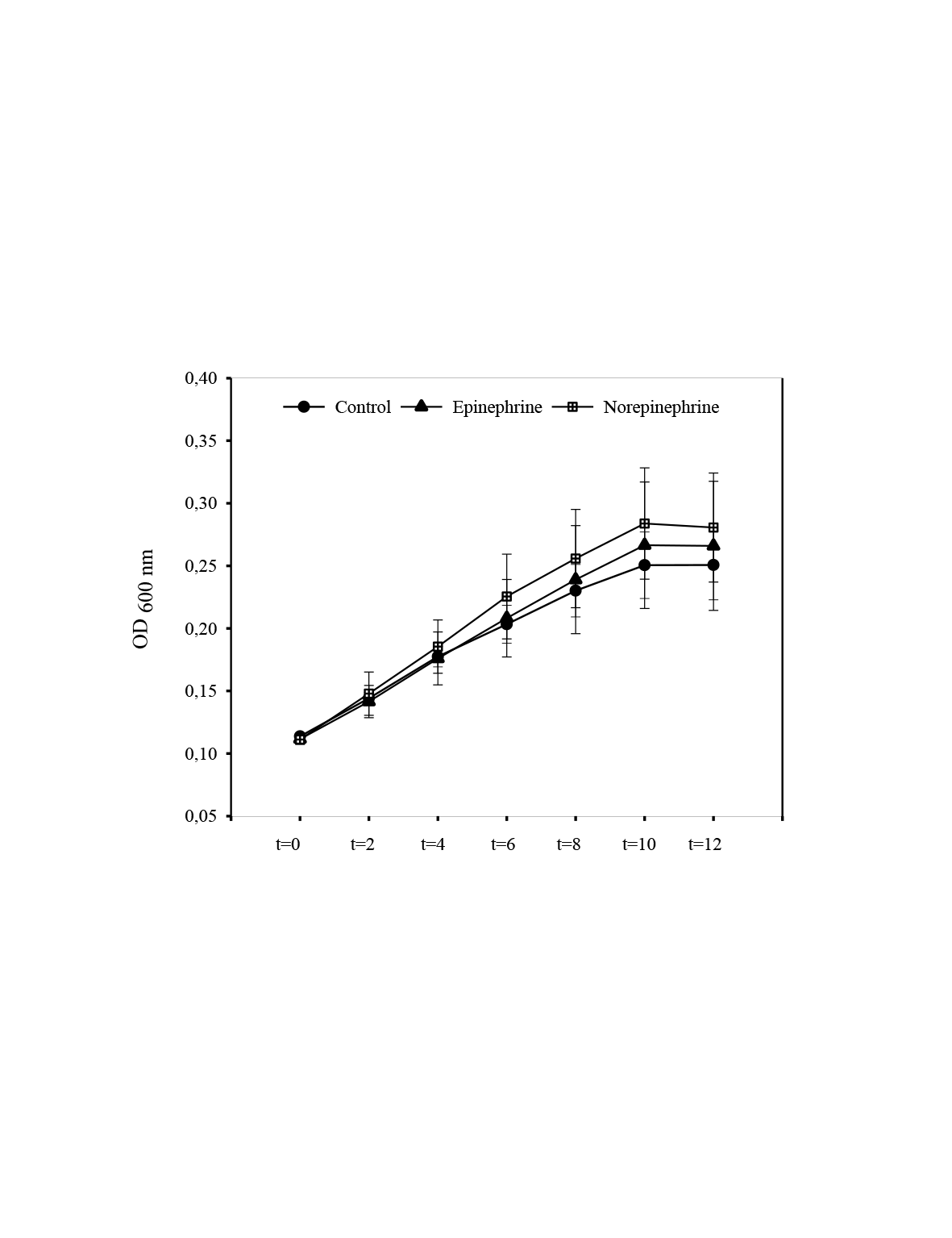

Supplement: S2 Fig — No significant effect on planktonic growth was observed in response to epinephrine or norepinephrine. (TIF) [file pone.0157334.s002.tif]
